# Circulating miR-10b-5p, miR-193a-3p, and miR-1-3p Are Deregulated in Patients with Heart Failure and Correlate with Hormonal Deficiencies

**DOI:** 10.3390/ijms26115225

**Published:** 2025-05-29

**Authors:** Anna Maria Grimaldi, Roberta D’Assante, Francesco Fiore, Simone Marcella, Stefania Paolillo, Francesco Cacciatore, Valentina Mercurio, Eduardo Bossone, Antonio Cittadini, Carlo Gabriele Tocchetti, Mariarosaria Incoronato

**Affiliations:** 1 IRCCS SYNLAB SDN, Via Emanuele Gianturco 113, 80143 Naples, Italy; annamaria.grimaldi@synlab.it (A.M.G.); s.marcella92@gmail.com (S.M.); 2 Department of Translational Medical Sciences, Federico II University, 80131 Naples, Italy; roberta.dassante@outlook.it (R.D.); francescocarta93@gmail.com (F.F.); francesco.cacciatore67@gmail.com (F.C.); valemercurio@yahoo.com (V.M.); antonio.cittadini@unina.it (A.C.); cgtocchetti@gmail.com (C.G.T.); 3 Department of Advanced Biomedical Sciences, Federico II University, 80131 Naples, Italy; paolilloste@gmail.com; 4 Interdepartmental Center of Clinical and Translational Sciences (CIRCET), Federico II University, 80131 Naples, Italy; 5 Interdepartmental Hypertension Research Center (CIRIAPA), Federico II University, 80131 Naples, Italy; 6 Department of Public Health, University Federico II of Naples, Via Sergio Pansini 5, 80131 Naples, Italy; 7 Center for Basic and Clinical Immunology Research (CISI), Federico II University, 80131 Naples, Italy

**Keywords:** heart failure, multiple hormonal deficiency, circulating miRNA, blood, plasma

## Abstract

Heart failure (HF) is among the most important causes of worldwide morbidity, hospitalisation, and mortality. A reduction in anabolic hormonal axes seems to potentially play an important role in chronic HF progression and prognosis. Several lines of evidence support the critical roles of miRNAs in the endocrine system, and differentially expressed miRNA patterns were found to be able to detect HF. To date, the ability of miRNAs to detect HF patients affected by hormonal deficiencies has yet to be addressed. The aim of this study was to explore the association between circulating miRNA profiles and multiple hormonal deficiencies in HF patients to provide new insights into HF pathophysiology. The study cohort included 129 subjects (94 HF patients and 35 controls). Circulating miRNAs assayed in plasma samples were miR-1-3p, miR-10b-5p, miR-24-3p, miR-193a-5p, miR-454-3p, miR-503-5p, miR-551b-3p, and miR-598-3p. NT-proBNP, IGF-1, fT3, DHEA-S, testosterone, HF subtypes, and NYHA class were also evaluated. A multiple hormonal deficiency syndrome (MHDS) was defined as the presence of ≥two hormone deficiencies. We found that miR-10b-5p, miR-193a-5p, and miR-1-3p could distinguish chronic HF patients from controls. The identified miRNAs were downregulated in HF patients, particularly those with NYHA I-II classifications and pathological values of NT-proBNP. In addition, these three circulating miRNAs correlated with each other, and their deregulation seems to be influenced by hormone deficiencies, especially in patients with reduced ejection fraction. Among the three miRNAs, miR-10b-5p was the best able to diagnose chronic HF-MHDS patients (AUC = 0.8). These results support the clinical utility of miR-10b-5p, miR-193a-5p, and miR-1-3p in detecting HF patients, especially those with hormone deficiencies.

## 1. Introduction

Heart failure (HF) is one of the most important causes of morbidity, hospitalisation, and mortality worldwide [1]. The neurohormonal system plays a crucial role in the induction and progression of HF, serving as the primary target for traditional heart failure medications [2]. Multiple hormonal and metabolic deficiency syndrome (MHDS) was identified as a condition characterised by two or more established alterations in anabolic hormones [hormone-insulin growth factor 1 (IGF-1) axis, serum testosterone, dehydroepiandrosterone sulfate (DHEA-S), free-triiodothyronine and free thyroxine (fT3 and fT4) levels] [3,4]. It has been observed that anabolic hormonal deficiencies (HDs) involving GH/IGF-1, thyroid hormones, testosterone, and DHEA-S, along with insulin resistance and type 2 diabetes mellitus, may coexist with chronic heart failure (CHF) [5,6]. In fact, Cittadini and colleagues [7] reported that the coexistence of at least two HDs is very common in HF patients. They also discovered a strong correlation between MHDS and a poor prognosis regarding the combined endpoint of all-cause mortality and cardiovascular hospitalisations in HF patients.

MicroRNAs (miRNAs) are small (∼22 nucleotides long) endogenous non-coding RNA molecules that regulate post-transcriptional gene expression by inhibiting the translation of mRNAs or inducing their degradation [8,9]. As miRNAs are particularly stable in biological fluids, deregulated across a wide range of diseases, and detected using simple laboratory methods, miRNAs are strong candidates for disease biomarkers [10,11,12,13]. miRNAs are known to play a crucial role in inflammatory conditions, including cancer, autoimmune disorders, neurodegenerative syndromes, and cardiovascular processes [14,15,16,17,18,19,20]. Distinct patterns of differentially expressed miRNAs have been linked to several pathophysiological mechanisms involved in HF, including cardiac remodelling, hypertrophy, apoptosis, fibrosis, and hypoxia [21,22,23,24,25]. Furthermore, multiple lines of evidence underscore the crucial roles of miRNAs in the endocrine system, regulating hormone production, activity, and target cell responsiveness [26]. Other studies have highlighted the ability of miRNAs to alter endocrine functions in patients with heart failure [27,28,29,30]. Overall, these data suggest that miRNAs are key molecules involved in deregulating the anabolic axis in patients with HF.

Recently, Wong and colleagues [25] successfully conducted a study to identify a circulating miRNA signature for the detection and subtyping of HF patients. The authors developed and validated two 8-miRNA panels as a diagnostic tool for detecting heart failure (HF) and discriminating between heart failure with reduced ejection fraction (HFrEF) and heart failure with preserved ejection fraction (HFpEF) subtypes. Their results established the efficacy of circulating miRNAs as diagnostic biomarkers for HF, demonstrating performance comparable to NT-proBNP. The model for HF detection included an miRNA signature (miR-24-3p, miR-454-3p, miR-551b-3p, miR-10b-5p, miR-503-5p, miR-193a-5p, and miR-598-3p) that, when coupled with NT-proBNP, enhanced their diagnostic capability. The strengths of their study were the relatively large number of HF patients (903) and control subjects (807), alongside two sizeable independent cohorts. Importantly, miR-1-3p has emerged as a promising candidate biomarker for monitoring muscular health, as it may be dysregulated in individuals with CHF and sarcopenia [31,32] and was found to be deregulated in serum samples of HF patients [33]. As the ability of miRNAs to detect HF patients affected by MHDS has yet to be addressed, and hormone deficiencies might be involved in the progression of HF [7], we utilised the miRNAs mentioned above to investigate whether their dysregulation in HF patients could be associated with deficiencies in hormonal axes.

## 2. Results

### 2.1. Circulating miRNA Expression in CHF Patients

**Figure 1:**
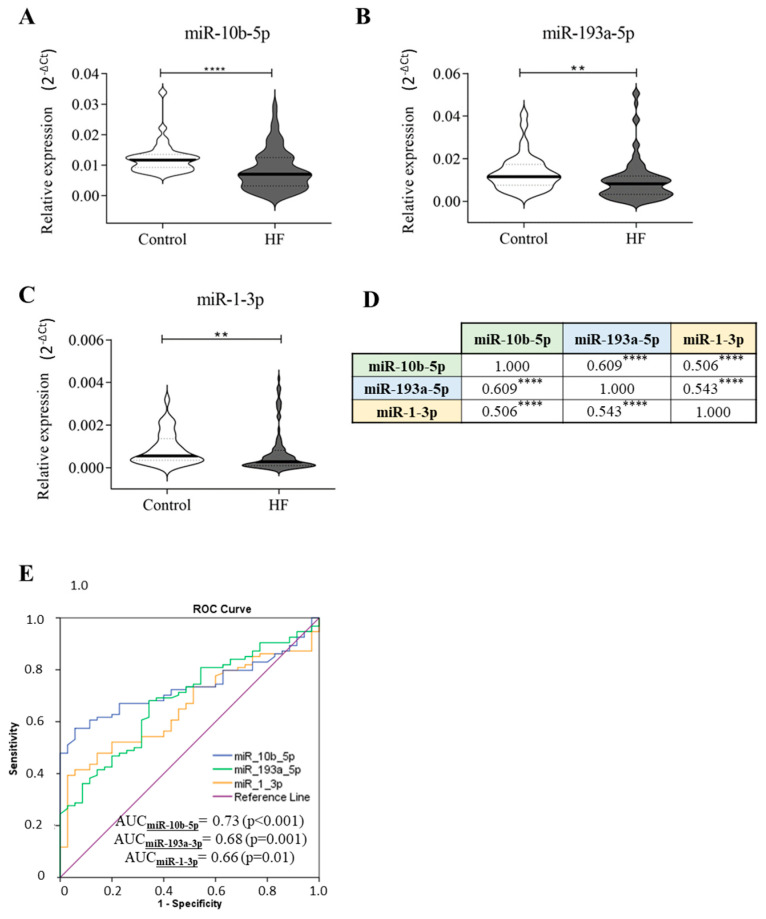
Circulating miRNA expression in HF patients and control subjects. Plasma samples of healthy subjects (*n* = 35) and HF patients (*n* = 94) have been screened for the presence of miR-24-3p, miR-454-3p, miR-551b-3p, miR-10b-5p, miR-503-5p, miR-193a-5p, miR-598-3p, and miR-1-3p. The relative expression was calculated as 2^−ΔCt^. Here, we show only the significant results: (**A**) miR-10b-5p, (**B**) miR-193a-5p, (**C**) miR-1-3p. (**D**) Correlation analyses. (**E**) ROC curve analyses and AUC values of the indicated miRNAs for discriminating healthy controls (*n* = 35) from HF patients (*n* = 94). The star symbol indicates statistical significance (**: *p* ≤ 0.01, ****: *p* ≤ 0.0001).

First, we evaluated the levels of several miRNAs that have been previously reported to be deregulated in HF [25,31]. We found that in our study cohort, only miR-10b-5p, miR-193a-5p, and miR-1-3p levels were significantly downregulated in the plasma samples of HF patients compared to control subjects (Figure 1A–C), and their trend was in line with those of Wong [25] and Sygitowicz [33]. There were no statistically significant differences in age and sex between HF patients and controls. Subsequently, correlation analyses were conducted, and as detailed in Figure 1D, the deregulation of these three miRNAs in HF patients strongly correlated with each other. Based on this finding, a receiver operating characteristic (ROC) curve analysis was performed. As shown in Figure 1E, we found that miR-10b-5p, miR-193a-5p, and miR-1-3p could discriminate HF patients from control subjects with areas under the curve (AUCs) of 0.73, 0.68, and 0.66, respectively.

Then, the diagnostic accuracy of each miRNA was evaluated. To this end, the cut-off values for miR-10b-5p, miR-193a-5p, and miR-1-3p were calculated using the “Youden index” statistic; the cut-off value defined for miR-10b-5p was 0.0076, for miR-193a-5p was 0.0106, and for miR-1-3p was 0.0002. Among the analysed molecules, miR-10b-5p and miR-193a-5p exhibited the highest diagnostic accuracy (Table 1). We optimised their diagnostic capability by evaluating a combined detection of these miRNAs. As reported in Table 1, the combination of the diagnostic test results for miR-10-3p and miR-193a-5p enhanced the accuracy (AUC = 0.72) compared to the evaluation of these molecules individually (AUC = 0.67). The combined analysis of all three miRNAs did not further increase the accuracy (AUC = 0.72).

### 2.2. miRNA Profiles and Clinical/Functional Indexes

#### 2.2.1. NT-proBNP, Ejection Fraction, Atrial Fibrillation, and Body Mass Index

**Figure 2:**
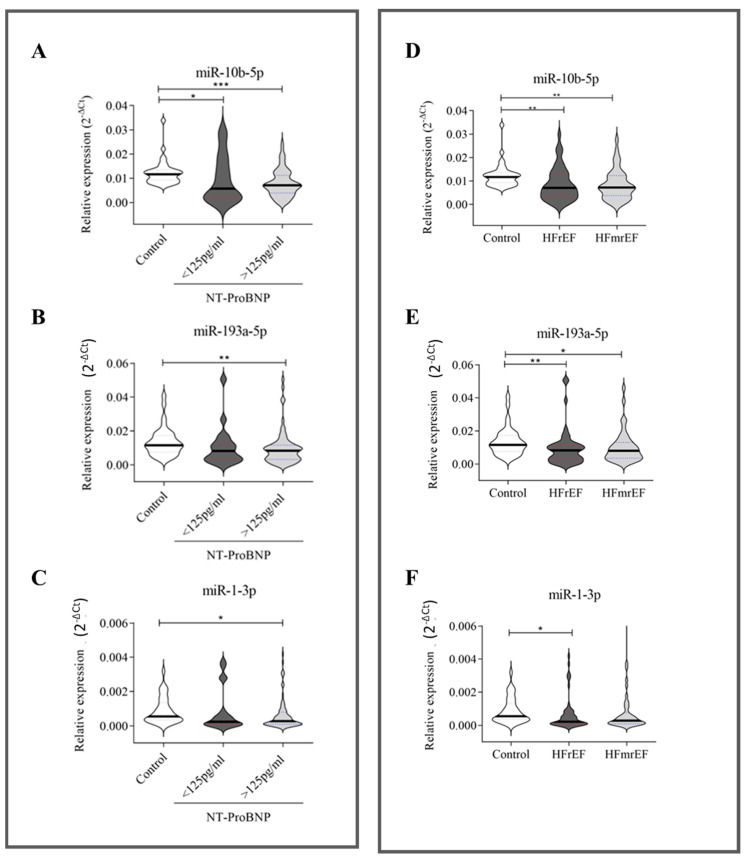
Relative expression of circulating miRNAs related to NT-proBNP and ejection fraction. NT-proBNP (**A**–**C**): 35 healthy subjects, 19 HF patients with NT-proBNP < 125 pg/mL, and 75 HF patients with NT-proBNP ≥ 125 pg/mL. Ejection fraction (**D**–**F**): healthy subjects (*n* = 35) and HF patients (*n* = 94) stratified according to ejection fractions—49 HF patients with reduced ejection fraction (HFrEF) and 45 HF patients with mildly reduced ejection fraction (HFmrEF). The relative expression was calculated as 2^−ΔCt^. The star symbol indicates statistical significance (*: *p* ≤ 0.05, **: *p* ≤ 0.01, ***: *p* ≤ 0.001).

We then stratified our HF patients according to their NT-proBNP values: 19 HF patients had NT-proBNP levels < 125 pg/mL, while 75 HF patients had NT-proBNP values ≥ 125 pg/mL (Supplementary Table S1). All three investigated miRNAs were associated with higher serum levels of NT-proBNP (≥125 pg/mL) (Figure 2, left panel). Furthermore, decreasing circulating miR-10b-5p was also weakly associated with NT-proBNP values < 125 pg/mL (Figure 2A).

Our HF cohort (Table 2) included 52.1% of patients with HFrEF and 47.9% with HFmrEF (Supplementary Table S2). As shown in Figure 2 (right panel), the downregulation of miR-10b-5p (Figure 2D) and miR-193a-5p (Figure 2E) was independent of EF. In contrast, the downregulation of miR-1-3p (Figure 2F) distinguished only HFrEF patients from healthy donors.

Additionally, we investigated whether comorbidities such as atrial fibrillation or obesity impacted miRNA levels in HF patients; however, no significant results were found.

#### 2.2.2. NYHA

**Figure 3:**
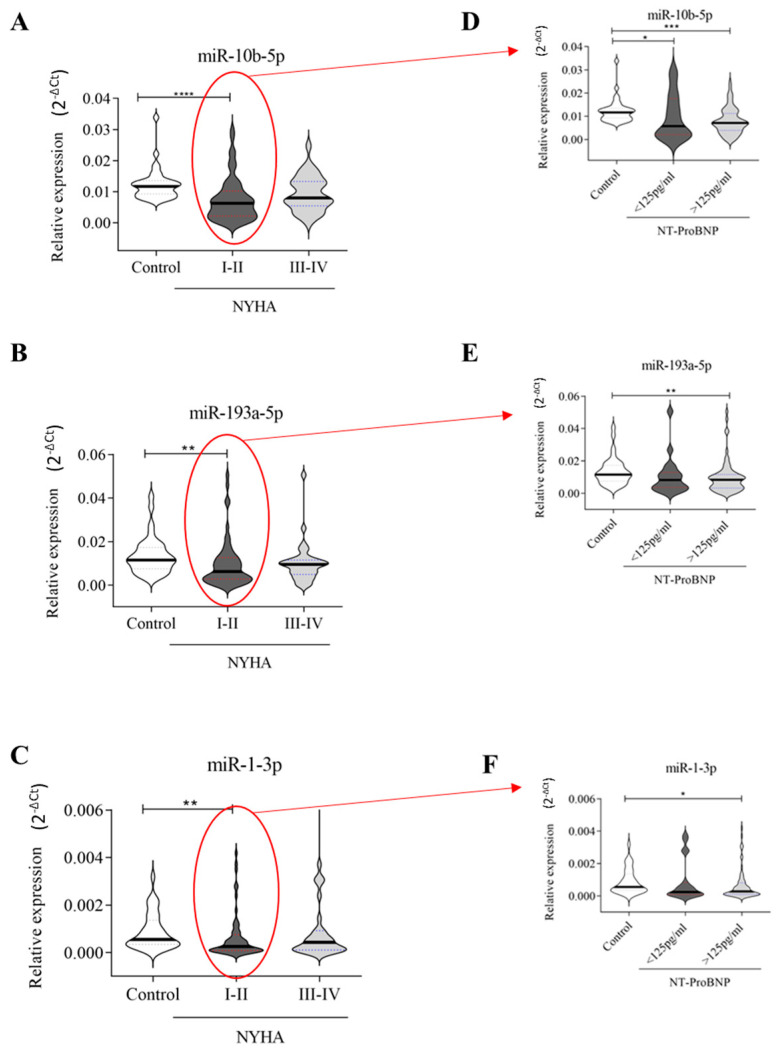
Relative expression of circulating miRNAs related to NYHA: (**A**–**C**) 35 healthy subjects, 60 HF patients with NYHA I-II, and 34 with NYHA III-IV; (**D**–**F**) HF patients with NYHA I-II were grouped according to NT-ProBNP values: 19 with NT-proBNP values < 125 pg/mL and 41 with NT-proBNP values ≥ 125 pg/mL. The relative expression was calculated as 2^−ΔCt^. The star symbol indicates statistical significance (*: *p* ≤ 0.05, **: *p* ≤ 0.01, ***: *p* ≤ 0.001, ****: *p* ≤ 0.0001). The relative expression was calculated as 2^−ΔCt^.

HF patients were classified based on NYHA (New York Heart Association) classifications into the I-II group (*n* = 60) and the III-IV group (*n* = 34) (Supplementary Table S3). Interestingly, the downregulation of all three miRNAs maintained its significance only in patients with NYHA class I-II compared to healthy donors (Figure 3A–C). It is known that the prognosis for patients with HF can be predicted by assessing the levels of NT-proBNP, the NYHA classification, and associated comorbidities [34]. Then, HF patients classified as NYHA I-II (*n* = 60) were further divided based on NT-proBNP values into those with <125 pg/mL (*n* = 19) and those with ≥125 pg/mL (*n* = 41). As reported in Figure 3D–F, we found that the downregulation of miR-10b-5p, miR-19a-5p, and miR-1-3 was significant (*p* ≤ 0.01) only in HF patients with pathological NT-proBNP values (≥125 pg/mL) when compared to healthy subjects, although miR-10b-5p was also weakly downregulated (*p* ≤ 0.05) in patients with NT-proBNP < 125 pg/mL in comparison to healthy subjects.

### 2.3. miRNA Profiles and Hormonal Axis Deficits

**Figure 4:**
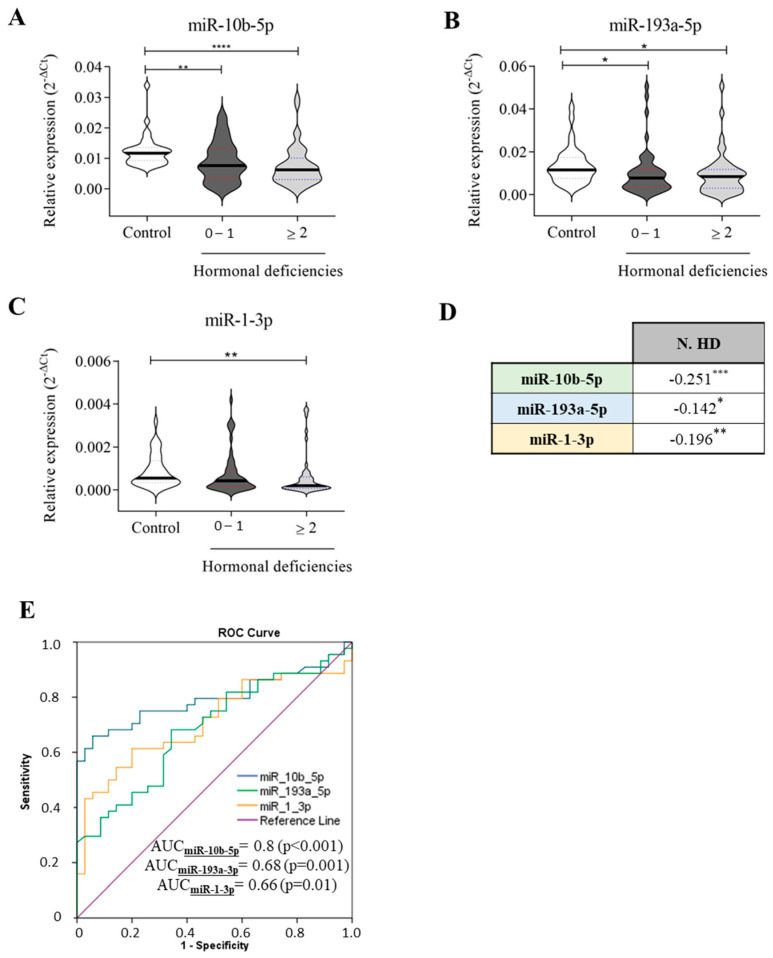
Relative expression of circulating miRNAs related to the number of hormonal deficiencies. The expression of (**A**) miR-10b-5p, (**B**) miR-193a-3p, and (**C**) miR-1-3p in healthy subjects (*n* = 35) and HF patients (*n* = 94) is stratified based on the number of hormonal deficiencies: 50 HF patients with 0–1 hormonal deficits and 44 HF patients with ≥2 hormonal deficits. (**D**) Correlation analyses between each miRNA and the number of hormone deficiencies (N. HD). (**E**) ROC curve analyses and AUC values of the indicated miRNAs for discriminating healthy controls (*n* = 35) from HF patients with hormonal deficiencies ≥ 2 hormones (*n* = 44). The relative expression was calculated as 2^−ΔCt^. The star symbol denotes statistical significance (*: *p* ≤ 0.05, **: *p* ≤ 0.01. ***: *p* ≤ 0.001, ****: *p* ≤ 0.0001).

MHDS encompasses several anabolic systems that are downregulated or impaired in HF, such as IGF-1, testosterone, DHEA-S, and fT3. Cittadini and colleagues previously demonstrated that MHDS impacts HF progression and outcome [7]. According to hormonal status, HF patients were categorised as follows: group 1 included those with 0-1 HD (NO-MHDS = 50), and group 2 comprised HF patients with HD ≥ 2 (MHDS = 44) (Supplementary Table S4). As illustrated in Figure 4, miR-10b-5p (A) and miR-193a-5p (B) were significantly downregulated in both groups compared to healthy controls. Nevertheless, miR-10b-5p downregulation more effectively detects (*p*-value ≤ 0.0001) MHDS rather than NO-MHDS (0–1 HD) patients when compared to healthy subjects. miR-1-3p was significantly downregulated only in MHDS patients (Figure 4C); in NO-MHDS, the circulating levels of miR-1-3p did not differentiate HF from healthy subjects. Interestingly, all three miRNAs correlated with the number of hormone deficits (Figure 2D), although miR-10-5p remained the molecule whose variation in expression was confirmed by the highest significance value. Therefore, a ROC curve analysis was recalculated to evaluate the ability of the three miRNAs to detect HF-MHDS patients (HD ≥ 2). Intriguingly, the ROC curve analysis results (Figure 2E) suggested that (i) miR-10-5p discriminated HF-MHDS patients from healthy subjects with a diagnostic accuracy that increased to 80% (compare Figure 2E, AUC = 0.73 with Figure 4E, AUC = 0.8); (ii) as the relative expression of miR-1-3p significantly discriminates only MHDS patients from healthy subjects (Figure 4C), the diagnostic ability of miR-1-3p was entirely attributable to the burden of MHDS patients within the total CHF cohort (compare Figure 1E, AUC = 0.66 with Figure 4E, AUC = 0.66).

**Figure 5:**
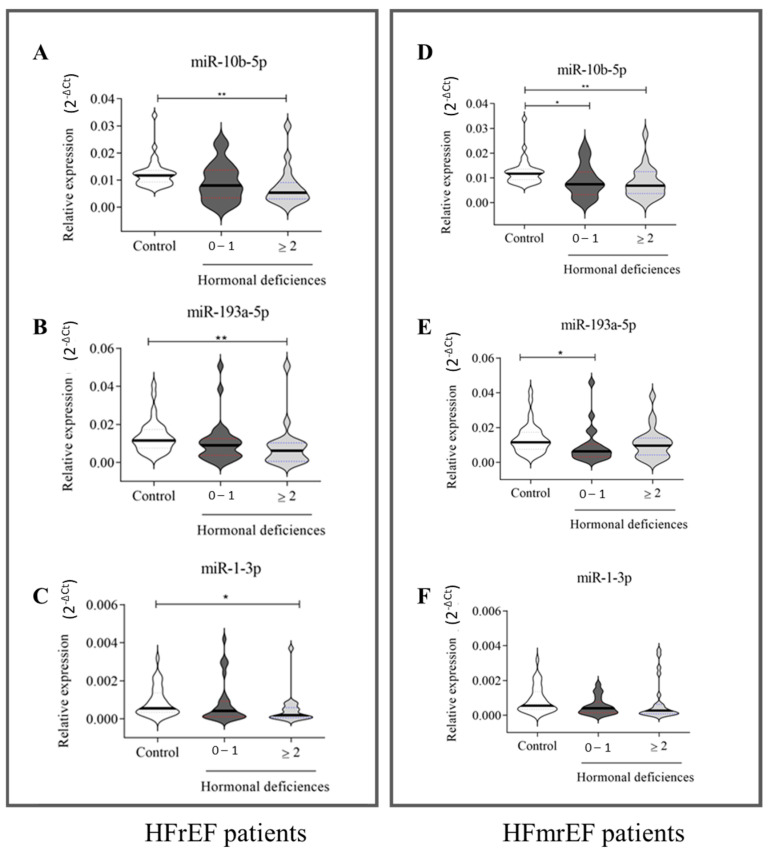
Relative expression of circulating miRNAs in HFrEF and HFmrEF patients grouped according to the number of hormonal deficits. HFrEF patients (**A**–**C**) and HFmrEF patients (**D**–**F**) are grouped based on the number of hormonal deficits. (**A**–**C**): A total of 35 healthy subjects, 29 HF patients with 0–1 hormonal deficits, and 20 HF patients with ≥2 hormonal deficits; (**D**–**F**): 35 healthy subjects, 21 HF patients with 0–1 hormonal deficits, and 24 HF patients with ≥2 hormonal deficits. The relative expression was calculated as 2^−ΔCt^. The star symbol indicates statistical significance (*: *p* ≤ 0.05, **: *p* ≤ 0.01).

We subsequently examined the trend of the three circulating miRNAs that stratify HFrEF and HFmrEF patients based on the number of hormonal deficiencies. Results in Figure 5 (left panel) indicate that miR-10b-5p (A), miR-193a-3p (B), and miR-1-3p (C) significantly detected only HFrEF patients with MHDS (HD ≥ 2). Conversely, in HFmrEF patients (Figure 5, right panel), miR-10b-5p (D) was significantly downregulated in HF patients regardless of the presence of HDs. Interestingly, miR-193a-5p (Figure 4E) was significantly downregulated only in patients with NO-MHDS (0–1 HD). No significant differences in miR-1-3p levels were observed between healthy subjects and HFmrEF patients (Figure 5F).

Furthermore, HF patients were stratified based on only one type of hormone deficiency to assess whether this single hormone deficit could impact the downregulation of miR-10b-5p, miR-193a-3p, and miR-1-3p. Nevertheless, no significant results were found.

### 2.4. miRNA and Gene Network Analysis

**Figure 6:**
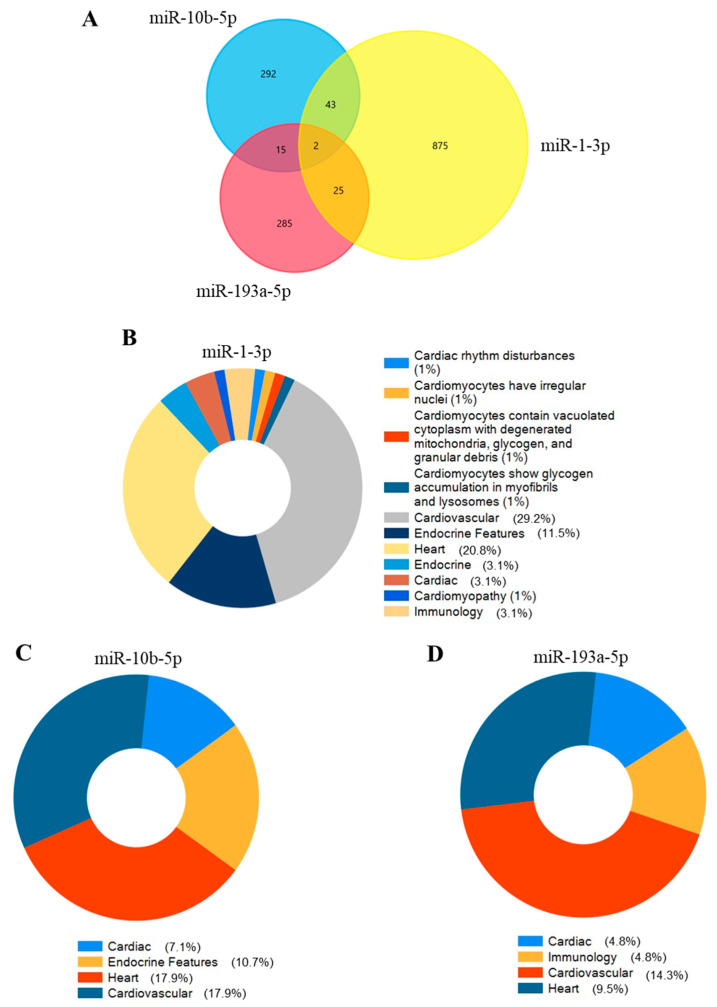
The miRNA-gene network for selected miRNAs and their target genes. (**A**) displays the number of shared and unique target genes for miR-1-3p, miR-10b-5p, and miR-193a-5p. Pie plots represent cardiovascular, endocrine, and immunology-related diseases that involve the target genes of (**B**) miR-1-3p, (**C**) miR-10b-3p, and (**D**) miR-193a-5p. Percentages do not sum to 100% because we included in the pie plots only relevant selected categories for our study. So, others unrelated or less relevant to our study were excluded.

The hypothetical functions of three selected miRNAs were assessed using the miRDB database to identify the target mRNAs. miR-1-3p, miR-10b-5p, and miR-193a-5p2 were associated with 945, 342, and 327 mRNAs in the miRDB database, respectively (Figure 6A). By querying FunRich (3.1.4), a software tool for gene enrichment analysis, we explored whether the target genes of each miRNA could be involved in cardiovascular, endocrine, and immunology disease classes, excluding all other possible diseases. The target genes of miR-1-3p were found to be involved in 29.2% of cardiovascular diseases (Figure 6B), whereas for mir-10b-5p, the percentage was 17.9% (Figure 6C), and for miR-193-3p, it was 14.3% (Figure 6D). Interestingly, the hypothetical gene targets of miR-10b-5p and miR-1-3p are also involved in endocrine diseases: 10.7% and 11.5%, respectively.

## 3. Discussion

miRNAs regulate gene expression and cellular functions. Their deregulation has been linked to multiple human conditions, including cardiovascular diseases [35]. As MHDS affects HF progression and outcomes [7], this study wants to explore the clinical relevance of the association between circulating miRNAs profiles and deficits in anabolic axes (single or multiple) in HF patients in order to provide new insights into HF pathophysiology. Therefore, we focused on miRNAs that the literature had already identified as correlated with HF [25,33] to investigate whether their deregulation was affected by hormone deficits.

We have presented results indicating that miR-10b-5p, miR-193a-5p, and miR-1-3p were downregulated in the plasma of HF patients, and their trend was consistent with those of Wong [25] and Sygitowicz [33]. Furthermore, their deregulation was strongly correlated, and the highest diagnostic accuracy was achieved by combining the miR-10b-5p with the miR-193a-5p (AUC = 0.72). Compared to the work of Wong and colleagues [25], our results were partially discrepant, as the remaining miRNAs we screened (miR-24-3p, miR-454-3p, miR-503-5p, miR-551b-3p, and miR-598-3p) were not deregulated in our study cohort under our experimental conditions. Nevertheless, the differences in sample size and ethnicity may account for this variation.

Therefore, we investigated whether the downregulation of miR-10b-5p, miR-193a-5p, and miR-1-3p may be associated with clinical and functional indices (NT-proBNP, HF subtypes, and NYHA classification). In clinical practice, NT-proBNP is a well-established biomarker recommended for risk stratification in individuals with chronic HF: the higher the concentration, the more severe the condition [36]. By stratifying HF patients according to NT-proBNP results (<125 pg/mL and ≥125 pg/mL), we found that the downregulation of miR-10b-5p, miR-193a-5p, and miR-1-3p was inversely associated with increasing values of NT-proBNP. Conversely, aside from miR-1-3b, the downregulation of miR-10b-5p and miR-193a-3p appeared to be unrelated to the HF subtype (HFrEF and HFmrEF).

As NT-proBNP levels reflect the severity of stable chronic HF expressed by the NYHA functional classification [34], we grouped our cohort according to class I-II and III-IV. Interestingly, we found that the three miRNAs were significantly associated with NYHA functional class I-II. In this class, we noted a significant association between miRNAs and higher values of NT-proBNP.

Furthermore, when we assayed the serum samples of HF patients and healthy subjects for IGF-1, testosterone, DHEA-S, and fT3, we observed an inverse correlation of the three miRNAs with the number of hormone deficits. Specifically, the downregulation of miR-1-3p in chronic HF patients was associated with the presence of ≥2 hormone deficits (MHDS), and the lowest level of circulating miR-10b-5p had the highest statistical significance in discriminating chronic HF-MHDS patients from healthy subjects. In support, the ROC curve analysis results showed that the ability of miR-10b-5p to diagnose chronic HF-MHDS patients was greater (AUC = 0.8) compared to its ability to detect only HF patients [regardless of the presence/absence of hormone deficit (AUC = 0.73)]. To the best of our knowledge, this is the first study that explores and identifies a link between the deregulation of miRNAs for HF diagnosis and hormone deficiencies.

Bioinformatic analyses were also conducted to enhance our understanding of these miRNAs’ physiological roles in cardiovascular and endocrine diseases. Our in silico studies revealed that the potential targets of miR-10b-5p, miR-193a-5p, and miR-1-3p may play a role in cardiologic and endocrine diseases. Among the three miRNAs analysed, miR-1-3p is the most extensively studied in myocardial infarction (MI) and heart failure [37]. In contrast, little is known about the roles of miR-10-b-5p and miR-193 in cardiological and hormonal pathological conditions. Morales-Sanchez and co-workers observed that nearly 50% of the disease annotations in which miR-1-3p could be involved were related to the cardiovascular system and endocrine disorders [38]. It has been suggested that miR-1 deregulation may be responsible for adverse structural remodelling of the heart [39], and its monitoring could prove beneficial in assessing the risk of secondary cardiovascular issues [39]. It was found to be lower in both acute and chronic HF patients compared to healthy individual controls [40] and combined with other circulating miRNAs, it was defined as a biomarker for acute myocardial infarction (AMI) [41]. Furthermore, IGF-1 protein and the IGF-1 receptor are targets of miR-1, with their expression being inversely correlated with IGF-1 protein levels in models of cardiac hypertrophy [41].

According to our data, the circulating levels of miR-10b-5p were lower in patients with advanced HF, and functional studies indicated that its downregulation, in conjunction with other circulating miRNAs, might be linked to clinical outcomes [42]. In addition, both in vivo and in vitro experiments demonstrated that the overexpression of mir-10b-5p reduced hypoxia-induced cardiomyocyte apoptosis, emphasising its protective role for the myocardium and its potential therapeutic applications in myocardial infarction [43]. In vitro and in vivo experiments displayed that the total amount of miR-193a-5p in AMI-Exosomes (circulating exosomes from AMI patients) was lower compared to Normal-Exosomes (healthy controls), and the ectopic transfection of miR-193a-5p into HUVEC cells facilitated the repair of endothelial damage [44]. Vitamin D3 deficiency, obesity, and diabetes mellitus increase the risk of cardiovascular diseases. A recent study demonstrated that the expression of miRNA-193a-5p was significantly lower in plasma samples from patients with diabetes mellitus, vitamin D deficiency, and obesity compared to the control patients [45]. Additionally, miR-193a-5p was found to regulate the IGF-1 signalling pathway by targeting the Phosphoinositide-3-Kinase Regulatory Subunit 3 (PIK3R3), which is involved in this pathway [41].

Our results support the clinical utility of miR-10b-5p, miR-193a-5p, and miR-1-3p in detecting HF patients. These new analyses and their relative results will significantly enhance our understanding of the pathophysiological aspects of this chronic disease. Our main goal was not only to define the diagnostic power of these miRNAs but also to evaluate whether the deregulation of these molecules could depend on specific clinical features that characterise HF patients (hormonal deficiencies in association with EF, NT-proBNP, and NYHA classes). The main limitation of this study was its small sample size, with limited ethnic diversity and disease etiologies, which may affect generalizability, particularly since our cohort was screened for four different hormones, rendering the MHDS group (≥two hormonal deficiencies) undoubtedly heterogeneous. Similarly, the downregulation of miRNAs was found to be significant in patients with NYHA I-II, and a larger study cohort would provide more robust results in distinguishing between NYHA I and II. Larger, multicentre studies are necessary to confirm and extend these findings. Furthermore, additional in vitro and in vivo studies are needed to improve mechanistic understanding and elucidate the relationship between these miRNAs and heart failure outcomes, especially in patients with hormonal deficiencies.

## 4. Materials and Methods

### 4.1. Patients and Study Design

Our prospective, multicentre study enrolled 94 HF patients in two years (2020–2022) from the Division of Internal Medicine and the Division of Cardiology of the Federico II University Hospital, Naples, Italy, recruited and followed according to this protocol [7], and 35 healthy control subjects, clinically healthy individuals with no known history of cardiovascular, endocrine, or systemic disease at the time of enrollment. Table 2 reports the clinical characteristics of the participants.

Inclusion criteria: Age > 18 years; ischemic or non-ischemic dilated cardiomyopathy; stable medications for at least three months, including β-blockers that had to be started at least 6 months before entering the study; LV ejection fraction (EF) below 50%; and signed informed consent.

Exclusion criteria: Active malignancy; unstable angina or recent myocardial infarction (within 6 months); severe kidney disease (serum creatinine levels > 2.5 mg/dL); inability to perform a cycle ergometer exercise test; diabetes mellitus in poor glycometabolic control (HbA1c > 8.5%) and/or proliferative retinopathy or severe non-proliferative retinopathy; advanced liver cirrhosis; ongoing neoplastic disease or history of malignancies; severe acute illness due to complications of open heart or abdominal surgery; multiple accidental trauma or acute respiratory failure; active infection or sepsis; and acute coronary syndrome in the previous 6 months.

All patients underwent a comprehensive medical history, an electrocardiogram, Doppler echocardiography, cardiopulmonary exercise stress testing (CPET) or a 6-min walking test (6MWT), and blood chemistry that included N-terminal pro-brain natriuretic peptide (NT-proBNP), IGF-1, fT3, DHEA-S, and testosterone. Blood samples were collected from all participants and assayed for circulating miRNA expression.

**Figure 7:**
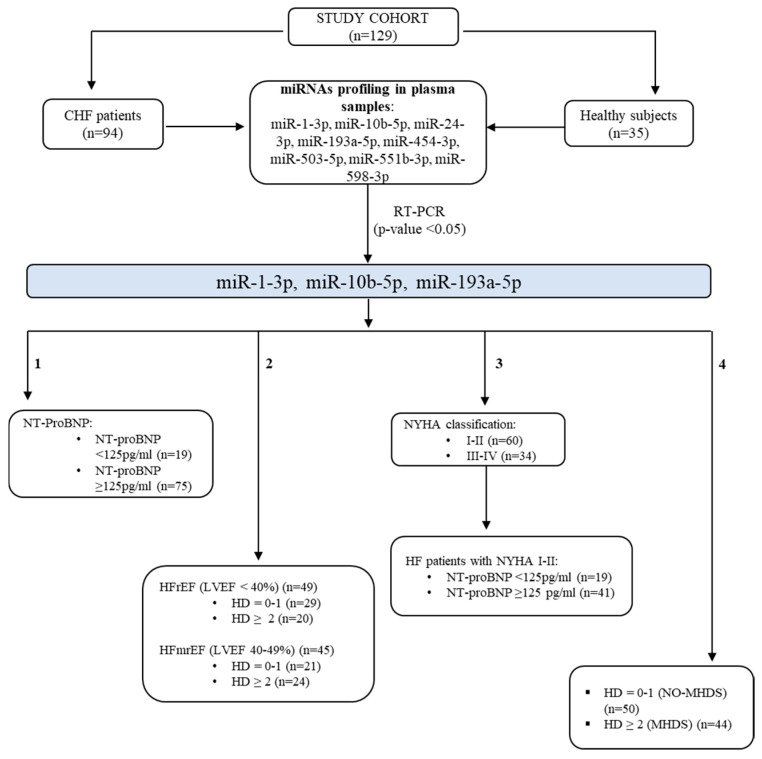
Study design. The study cohort (n.129 subjects) includes 94 chronic HF patients and 35 healthy donors. Eight miRNAs were screened in plasma samples. miR-10b-5p, miR-193a-5p, and miR-1-3p were significantly deregulated in HF patients (*p*-value < 0.05). Their deregulation was explored by grouping the chronic HF patients based on their clinical/functional characteristics as follows: (1) serum levels of NT-proBNP, (2) ejection fraction, (3) NYHA classification, and (4) the number of hormone deficiencies (HDs). HF: heart failure; MHDS: multiple hormonal deficiency syndrome; HFrEF: heart failure with reduced ejection fraction; HFmrEF: heart failure with mildly reduced ejection fraction; NT-ProBNP: N-terminal pro B-type natriuretic peptide; NYHA: New York Heart Association.

The study design is illustrated in Figure 7. Specifically, a signature of eight circulating miRNAs (miR-1-3p, miR-10b-5p, miR-24-3p, miR-193a-5p, miR-454-3p, miR-503-5p, miR-551b-3p, and miR-598-3p) identified as biomarkers for HF detection [25,31] was assessed for diagnostic capability in plasma samples from our study cohorts. Those statistically deregulated under our experimental conditions were analysed by stratifying patients according to the number of HD, subtypes, and NYHA classifications. The criteria for determining single hormone deficiency are described in Table 3. Patients were grouped based on the number of hormone deficiencies: 0–1 hormone deficiency (NO-MHDS) (*n* = 50) and two or more hormone deficiencies (MHDS) (*n* = 44). HF patients were classified according to their EF (HFrEF, *n* = 49; HFmrEF, *n* = 45) and their NYHA Functional classifications [2] as follows: classes I-II (*n* = 60) and classes III-IV (*n* = 34).

### 4.2. Plasma Collection, RNA Extraction, and Reverse Transcription

Plasma was obtained from whole blood samples through centrifugation at 1900× *g* for 10 min at 4 °C. The supernatant was centrifuged at 16,000× *g* for another 10 min at 4 °C and stored at the Biobank SDN [46] in aliquots of 0.5 mL at −80 °C until analysis. The extraction of total RNA from 200 μL of plasma was carried out using the miRNeasy Serum/Plasma Kit (Qiagen, Hilden, Germany) according to the manufacturer’s instructions. To provide a control for RNA isolation, cDNA synthesis, and PCR amplification for miRCURY LNA miRNA PCR, we utilised a Spike-in Kit (Qiagen) that included four Spike-in templates (cel-mir-39-3p, UniSp2, UniSp4, and Unisp5). Briefly, 1 μL of Spike-in templates was mixed with the lysis buffer before combining with the plasma. Total RNA (including miRNAs) was eluted in 14 μL of RNase-free water. Reverse transcription was carried out using the miRCURY LNA™ RT Kit (Qiagen, Hilden, Germany) following the manufacturer’s guidelines. In summary, 1 μL of UniSp6 spike-in (Qiagen, Hilden, Germany), serving as a control for the RT reaction’s quality, was incorporated into the reaction mix, which contained 2 μL of total RNA, nucleic acid mix buffer, and reverse transcriptase, resulting in a final volume of 10 μL. The RT mix was incubated for 60 min at 42 °C and 5 min at 95 °C. cDNA was stored at −20 °C until analysis.

### 4.3. Quantitative Real-Time PCR (qRT-PCR)

The expression values of miR24-3p, miR454-3p, miR551b-3p, miR10b-5p, miR503-5p, miR193a-5p, miR598-3p, and miR1-3p were determined by real-time PCR using miRCURY LNA miRNA primers (Qiagen, Hilden, Germany) and the miRCURY LNA™ SYBR Green PCR Kit (Qiagen, Hilden, Germany), with the instrument CFX384 (Biorad, California). The PCR cycling conditions included 95 °C for 2 min, followed by 40 cycles of 95 °C for 10 s and 56 °C for 60 s, along with melting curve analysis from 60 to 95 °C. The threshold cycle value for Real-Time PCR was set at 280. There are no definitive guidelines ruling data normalisation in miRNA expression analysis [47], so, according to our previous study [48], we used a spike-in control cel-miR-39-3p miRNA mimic. The Ct values of each miRNA were measured in triplicate, and the miRNA expression levels were calculated using the formula 2^−ΔCt^. The data are presented as median ± standard deviation.

### 4.4. Measurement of Blood Chemistry

The serology tests were conducted according to the manufacturer’s protocols and reference intervals. Specifically, NT-proBNP, fT3, and testosterone were measured using the Atellica^®^ Solution Immunoassay & Clinical Chemistry Analysers (Siemens Healthineers, Milano, Italy), while IGF-1 and DHEA-S were assessed with the IMMULITE^®^ 2000 XPi Immunoassay System (Siemens Healthineers). The threshold values provided by the supplying company are as follows: 125 pg/mL for NT-proBNP; 2.30–4.20 pg/mL for fT3; approximately 1.6–7.5 ng/mL for males under 50 years old and 0.9–7.9 ng/mL for males over 50 years old; and 25 ng/dL for females. Regarding DHEA-S, the values are 80–560 µg/dL for males and 35–430 µg/dL for females, respectively. Finally, concerning IGF-1, the ranges are as follows: 116–358 ng/mL for those aged 21–25, 117–329 ng/mL for those aged 26–30, 115–307 ng/mL for those aged 31–35, 109–284 ng/mL for those aged 36–40, 101–267 ng/mL for those aged 41–45, 94–252 ng/mL for those aged 46–50, 87–238 ng/mL for those aged 51–55, 81–225 ng/mL for those aged 56–60, 75–212 ng/mL for those aged 61–65, 69–200 ng/mL for those aged 66–70, 64–188 ng/mL for those aged 71–75, 59–177 ng/mL for those aged 76–80, and 55–166 ng/mL for those aged 81–85.

### 4.5. miRNA-Gene Network Analysis

The three miRNAs obtained were tested using the miRDB database http://mirdb.org (accessed on 27 August 2024) to predict the targeted genes [49]. FunRich http://www.funrich.org (accessed on 27 August 2024) was used to perform PPI networks and functional enrichment analysis [50].

### 4.6. Statistical Analysis

Data were analysed using GraphPad Prism 10.1.2 and SPSS statistic 20. Shapiro–Wilk analysis assessed the normality of the distribution for each parameter. The Mann–Whitney U and Kruskal–Wallis tests, followed by Dunn’s correction for multiple comparisons, evaluated the significance of differences between groups. Spearman’s rank-order correlation and Kendall’s rank correlation tests verified the correlations and associations among biological markers. The chi-square test and Fisher’s test analysed categorical variables.

Receiver operating characteristic (ROC) curves and the area under the ROC curve (AUC) were employed to assess each miRNA’s specificity, diagnostic capability, and sensitivity. The optimal cut-off values differentiating between healthy and CHF patients were calculated from the data distribution on the ROC curves. Subsequently, the Youden index (J = Sensitivity + Specificity − 1) was utilised to identify the most effective diagnostic miRNA with the highest sensitivity and specificity. ROC curves for individual markers and combinations of markers were computed in SPSS.

We evaluated the diagnostic accuracy of each circulating miRNA and their combinations. Statistical significance differences were accepted when the *p*-value was ≤0.05.

## 5. Conclusions

Two main findings emerged from our study. Firstly, miR-10b-5p, miR-193a-5p, and miR-1-3p are deregulated in HF patients, primarily those with NYHA I-II and elevated levels of NT-proBNP. Secondly, their deregulation seems to be influenced by hormone deficiencies, with the highest significance noted for miR-10b-5p. The clinical relevance of these findings necessitates further investigation, as a larger sample size is essential for validating these scientific results. Preclinical studies are required to explore the relationship between the identified miRNAs, HF, and hormone deficiencies.

## Figures and Tables

**Table 1:** Diagnostic accuracy of the indicated circulating biomarkers for discriminating healthy subjects from CHF patients.

miR-10b-5p	Test −	Test +	Total	Sensitivity %	Specificity %	Diagnostic Accuracy
Disease −	33 (TN)	2 (FP)	35	57.4	94.3	0.67
Disease +	40 (FN)	54 (TP)	94			
Total	73	56	129			
miR-193a-5p	Test −	Test +	Total	Sensitivity	Specificity	Diagnostic accuracy
Disease −	23 (TN)	12 (FP)	35	68.1	65.7	0.67
Disease +	30 (FN)	64 (TP)	94			
Total	53	76	129			
miR-1-3p	Test −	Test +	Total	Sensitivity	Specificity	Diagnostic accuracy
Disease −	34 (TN)	1 (FP)	35	36.2	97.1	0.53
Disease +	60 (FN)	34 (TP)	94			
Total	94	35	129			
miR-1-3p + miR-10b-5p	Test −	Test +	Total	Sensitivity	Specificity	Diagnostic accuracy
Disease −	32 (TN)	3 (FP)	35	62.8	91.4	0.7
Disease +	35 (FN)	59 (TP)	94			
Total	67	62	129			
miR-1-3p + miR-193a-5p	Test −	Test +	Total	Sensitivity	Specificity	Diagnostic accuracy
Disease −	22 (TN)	13 (FP)	35	72.3	62.9	0.7
Disease +	26 (FN)	68 (TP)	94			
Total	48	81	129			
miR-10b-3p + miR-193a-5p	Test −	Test +	Total	Sensitivity	Specificity	Diagnostic accuracy
Disease −	23 (TN)	12 (FP)	35	74.5	65.7	0.72
Disease +	24 (FN)	70 (TP)	94			
Total	47	82	129			
miR-1-3p + miR-10b-5p + miR-193a-5p	Test −	Test +	Total	Sensitivity	Specificity	Diagnostic accuracy
Disease −	22 (TN)	13 (FP)	35	75.5	62.9	0.72
Disease +	23 (FN)	71 (TP)	94			
Total	45	84	129			

Disease + (HF patients), Disease − (healthy subjects), true positive (TP), false positive (FP), false negative (FN), true negative (TN), and HF (heart failure).

**Table 2:** Clinical characteristics of HF patients.

Clinical Indexes	HF Patients (*n* = 94)
Age (years)	64 ± 12
Gender (*n*; % Male)	76; 81
NYHA I (*n*; %)	10; 11
NYHA II (*n*; %)	50; 53
NYHA III (*n*; %)	30; 32
NYHA IV (*n*; %)	4; 4
Etiology (*n*; % ischemics)	55; 59
Systolic blood pressure (mm/Hg)	123 ± 15
Diastolic blood pressure (mm/Hg)	75 ± 12
Type II diabetes mellitus, (*n*; %)	18; 19
BMI (Kg/m^2^)	28 ± 5
eGFR (mL/min per 1.73 m^2^)	70 ± 20
NT-proBNP (pg/mL)	1695 ± 3249
Left ventricular ejection fraction	38 ± 7
Atrial fibrillation (*n*; %)	17; 18
ICD (*n*; %)	27; 29
CRT (*n*; %)	17; 18
Drugs	
β-blockers (*n*; %)	51; 54
ACE-I/ARBs (*n*; %)	40; 43
MRA (*n*; %)	25; 27
Diuretics (*n*; %)	38; 40
Amiodarone (*n*; %)	12; 13
Digoxin (*n*; %)	3; 3
Antiplatelet drugs and/or anticoagulants (*n*; %)	50; 53
Statins (*n*; %)	44; 47
Ivabradine (*n*; %)	4; 4
Antidiabetics (*n*; %)	16; 17

Data are expressed as mean ± SD. HF, heart failure; NYHA, New York Heart Association; BMI, body mass index; eGFR, estimated glomerular filtration rate; NT-proBNP, N-terminal proB-type natriuretic peptide; ICD, implantable cardioverter-defibrillator; CRT, cardiac resynchronisation therapy; ACE-I, angiotensin-converting-enzyme; ARBs, angiotensin-receptor blockers; MRA, mineralocorticoid receptor antagonists.

**Table 3:** Definition of hormone deficiencies.

Hormone Deficiency	Value for Diagnosis
Insulin-like growth factor 1 (IGF-1)	age < 55 years: <122 ng/mL 55 years < age < 64.9 years: <109 ng/mL 65 years < age < 74.9 years: <102 ng/dL age > 75 years: <99 ng/dL
Testosterone	Men: <300 ng/dL Women: <25 ng/dL
Dehydroepiandrosterone sulfate (DHEA-S)	<80 µg/dL
Free triiodothyronine (fT3)	<2 pg/mL (3.1 mmol/L) with TSH in the normal range (0.55–4.78 µUI/mL)

## Data Availability

The data presented in this study are available on request from the corresponding author.

## Supplementary Materials

The following supporting information can be downloaded at: https://www.mdpi.com/article/10.3390/ijms26115225/s1.
