# Supplementary material for: Circulating miR-10b-5p, miR-193a-3p, and miR-1-3p Are Deregulated in Patients with Heart Failure and Correlate with Hormonal Deficiencies
Source: Int J Mol Sci. 2025 May 29;26(11):5225. doi: 10.3390/ijms26115225 (PMC12155496; doi:10.3390/ijms26115225)
Supplement: Supplementary file 1 [file ijms-26-05225-s001.zip › ijms-3662132-supplementary.pdf]

Table S1. Clinical characteristics of HF patients with NT-proBN<125 pg/ml and NT-proBNP ≥125pg/ml.

**Clinical indexes**	**HF patients with NT-proBNP <125pg/mL (*n*=19)**	**HF patients with NT-proBNP ≥125pg/mL (*n*=75)**	**p-value**
Age (years)	57 ± 12.1	66 ± 11	n.s.
Gender (Males, *n*; %)	14; 79	62; 81	n.s.
NYHA I (*n*; %)<br>NYHA II (*n*; %)<br>NYHA III (*n*; %)<br>NYHA IV (*n*; %)	3; 16<br>16; 84<br>0; 0<br>0; 0	7; 10<br>34; 45<br>30; 40<br>4; 5	n.s.<br>**0.0038**<br>**0.0003**<br>n.s.
Ischemic etiology (*n*; %)	10; 53	45; 60	n.s.
Systolic blood pressure (mm/Hg)	125 ± 22	122 ± 19	n.s.
Diastolic blood pressure (mm/Hg)	79 ± 12	73 ± 11	n.s.
Type II diabetes mellitus (*n*; %)	4; 21	14; 19	n.s.
BMI (Kg/m^2)	29 ± 6	30 ± 7	**0.0318**
eGFR (ml/min per 1.73m^2)	83 ± 8	66 ± 21	n.s.
Left ventricular ejection fraction	38 ± 9	36 ± 7	**0.0408**
Atrial fibrillation (*n*; %)	2; 10	15; 20	n.s.
ICD (*n*; %)	5; 26	22; 29	n.s.
CRT (*n*; %)	4; 21	13; 17	n.s.
Drugs (%)			
β-blockers (*n*; %)	10; 53	41; 55	n.s.
ACE-I/ARBs (*n*; %)	7; 37	33; 44	n.s.
MRA (*n*; %)	5; 26	20; 27	n.s.
Diuretics (*n*; %)	6; 32	32; 43	n.s.
Amiodarone (*n*; %)	0; 0	12; 16	n.s.
Digoxin (*n*; %)	0; 0	3; 4	n.s.
Antiplatelet drugs and/or anticoagulants (*n*; %)	10; 53	40; 53	n.s.
Statins (*n*; %)	10; 53	34; 45	n.s.
Ivabradine (*n*; %)	0; 0	4; 5	n.s.
Antidiabetics (*n*; %)	5; 26	11; 15	n.s.

Quantitative variables are expressed as mean ± SD. Categorical variables are expressed as numbers and percentages. HF, heart failure; with reduced ejection fraction; NYHA, New York Heart Association; BMI, body mass index; eGFR, estimated glomerular filtration rate; NT-proBNP, N-terminal proB-type natriuretic peptide; ICD, implantable cardioverter-defibrillator; CTR, cardiac resynchronization therapy; ACE-I, angiotensin-converting-enzyme; ARBs, angiotensin-receptor blockers; MRA, mineralocorticoid receptor antagonists; n.s., not significant. Bold values identify the statistical significance in the comparison of two groups (p-value <0.05).

Table S2. Clinical characteristics of HFrEF and HFmrEF patients.

**Clinical indexes**	**HFrEF (*n*=49)**	**HFmrEF (*n*=45)**	**p-value**
Age (years)	64 ± 13	64 ± 10	n.s.
Gender (Males, *n*; %)	33; 84	43; 78	n.s.
NYHA I (*n*; %)<br>NYHA II (*n*; %)<br>NYHA III (*n*; %)<br>NYHA IV (*n*; %)	1; 2<br>23; 47<br>23; 47<br>2; 4	9; 20<br>27; 60<br>7; 15<br>2; 5	**0.0061**<br>n.s.<br>**0.0017**<br>n.s.
Ischemic etiology (*n*; %)	27; 55	28; 62	n.s.
Systolic blood pressure (mm/Hg)	118 ± 20	128 ± 18	**0.0392**
Diastolic blood pressure (mm/Hg)	72 ± 12	77 ± 11	**0.0321**
Type II diabetes mellitus (*n*; %)	7; 14	10; 22	**0.0238**
BMI (Kg/m^2)	31 ± 6	30 ± 3	n.s.
eGFR (ml/min per 1.73m^2)	68 ± 20	76 ± 21	n.s.
NT-proBNP (pg/ml)	1763.7 ± 3977	1644 ± 2537	**0.0038**
Atrial fibrillation (*n*; %)	9; 18	8; 18	n.s.
ICD (*n*; %)	16; 33	11; 24	n.s.
CRT (*n*; %)	12; 24	5; 11	n.s.
Drugs			
β-blockers (*n*; %)	26; 53	25; 56	n.s.
ACE-I/ARBs (*n*; %)	18; 37	22; 49	n.s.
MRA (*n*; %)	16; 33	9; 20	n.s.
Diuretics (*n*; %)	23; 47	15; 33	**0.0239**
Amiodarone (*n*; %)	10; 20	2; 4	**0.0086**
Digoxin (*n*; %)	1; 2	2; 4	n.s.
Antiplatelet drugs and/or anticoagulants (*n*; %)	26; 53	24; 53	n.s.
Statins (*n*; %)	21; 43	23; 51	n.s.
Ivabradine (*n*; %)	3; 6	1; 2	n.s.
Antidiabetics (*n*; %)	9; 18	7; 16	n.s.

Quantitative variables are expressed as mean ± SD. Categorical variables are expressed as numbers and percentages. HFrEF, heart failure with reduced ejection fraction; HFmrEF, heart failure with mildly reduced ejection fraction; NYHA, New York Heart Association; BMI, body mass index; eGFR, estimated glomerular filtration rate; NT-proBNP, N-terminal proB-type natriuretic peptide; ICD, implantable cardioverter-defibrillator; CRT, cardiac resynchronization therapy; ACE-I, angiotensin-converting-enzyme; ARBs, angiotensin-receptor blockers; MRA, mineralocorticoid receptor antagonists; n.s., not significant. Bold values identify the statistical significance in the comparison of two groups (p-value <0.05).

Table S3. Clinical characteristics of HF patients with NYHA I-II and III-IV classification.

**Clinical indexes**	**NYHA I-II (*n*=60)**	**NYHA III-IV (*n*=34)**	**p-value**
Age (years)	64 ± 12	64 ± 12	n.s.
Gender (Males, *n*; %)	47; 78	29; 85	n.s.
Ischemic etiology (*n*; %)	35; 58	20; 59	n.s.
Systolic blood pressure (mm/Hg)	125 ± 21	120 ± 18	n.s.
Diastolic blood pressure (mm/Hg)	77 ± 13	72 ± 9	n.s.
Type II diabetes mellitus (*n*; %)	9; 15	9; 26	n.s.
BMI (Kg/m^2)	30 ± 4	31 ± 6	n.s.
eGFR (ml/min per 1.73m^2)	76 ± 18	59 ± 20	**0.0074**
NT-proBNP (pg/ml)	1397 ± 2408	2245 ± 4378	**0.0050**
Left ventricular ejection fraction	38 ± 6	39 ± 8	**0.0008**
Atrial fibrillation (*n*; %)	5; 8	12; 35	**0.068**
ICD (*n*; %)	17; 28	10; 29	n.s.
CRT (*n*; %)	10; 17	7; 21	n.s.
Drugs			
β-blockers (*n*; %)	30; 50	21; 62	n.s.
ACE-I/ARBs (*n*; %)	27; 45	13; 38	n.s.
MRA (*n*; %)	14; 23	11; 32	n.s.
Diuretics (*n*; %)	18; 30	20; 59	n.s.
Amiodarone (*n*; %)	3; 5	9; 26	**0.0070**
Digoxin (*n*; %)	2; 3	1; 3	n.s.
Antiplatelet drugs and/or anticoagulants (*n*; %)	31; 52	19; 56	n.s.
Statins (*n*; %)	30; 50	14; 41	n.s.
Ivabradine (*n*; %)	3; 5	1; 3	n.s.
Antidiabetics (*n*; %)	9; 15	7; 21	n.s.

Quantitative variables are expressed as mean ± SD. Categorical variables are expressed as numbers and percentages. HF, heart failure; NYHA, New York Heart Association; BMI, body mass index; eGFR, estimated glomerular filtration rate; NT-proBNP, N-terminal proB-type natriuretic peptide; ICD, implantable cardioverter-defibrillator; CTR, cardiac resynchronization therapy; ACE-I, angiotensin-converting-enzyme; ARBs, angiotensin-receptor blockers; MRA, mineralocorticoid receptor antagonists; n.s., not significant. Bold values identify the statistical significance in the comparison of two groups (p-value <0.05).

Table S4. Clinical characteristics of HF with 0-1 (NO-MHDS) and ≥ 2(MHDS) hormonal deficiencies.

**Clinical indexes**	**NO-MHDS (*n*=50)**	**MHDS (*n*=44)**	**p-value**
Age (years)	63 ± 11	66 ± 13	n.s.
Gender (Males, *n*; %)	43; 90	33; 70	n.s.
NYHA I (*n*; %)<br>NYHA II (*n*; %)<br>NYHA III (*n*; %)<br>NYHA IV (*n*; %)	6; 12<br>23; 46<br>18; 36<br>3; 6	4; 9<br>27; 61<br>12; 27<br>1; 3	n.s.<br>n.s.<br>n.s.<br>n.s.
Ischemic etiology (*n*; %)	29; 58	26; 59	n.s.
Systolic blood pressure (mm/Hg)	118 ± 17	129 ± 21	n.s.
Diastolic blood pressure (mm/Hg)	72 ± 10	78 ± 12	n.s.
Type II diabetes mellitus (*n*; %)	9; 18	9; 20	
BMI (Kg/m^2)	29 ± 5	31 ± 5	n.s.
eGFR (ml/min per 1.73m^2)	73 ± 19	65 ± 21	n.s.
NT-proBNP (pg/ml)	1544 ± 3794	1861 ± 2576	n.s.
Left ventricular ejection fraction	35 ± 8	36 ± 7	n.s.
Atrial fibrillation (*n*; %)	9; 18	8; 18	n.s.
ICD (*n*; %)	16; 32	11; 25	n.s.
CRT (*n*; %)	11; 22	6; 14	n.s.
Drugs			
β-blockers (*n*; %)	30; 60	21; 48	n.s.
ACE-I/ARBs (*n*; %)	21; 42	19; 43	n.s.
MRA (*n*; %)	16; 32	9; 20	n.s.
Diuretics (*n*; %)	22; 44	16; 36	n.s.
Amiodarone (*n*; %)	7; 14	5; 11	n.s.
Digoxin (*n*; %)	1; 2	2; 5	n.s.
Antiplatelet drugs and/or anticoagulants (*n*; %)	27; 54	23; 52	n.s.
Statins (*n*; %)	26; 52	18; 41	n.s.
Ivabradine (*n*; %)	2; 4	2; 5	n.s.
Antidiabetics (*n*; %)	7; 14	9; 20	n.s.

Quantitative variables are expressed as mean ± SD. Categorical variables are expressed as numbers and percentages. HF, heart failure; MHDS, multiple hormonal and metabolic deficiency syndrome; NYHA, New York Heart Association; BMI, body mass index; eGFR, estimated glomerular filtration rate; NT-proBNP, N-terminal proB-type natriuretic peptide; ICD, implantable cardioverter-defibrillator; CTR, cardiac resynchronization therapy; ACE-I, angiotensin-converting-enzyme; ARBs, angiotensin-receptor blockers; MRA, mineralocorticoid receptor antagonists; n.s., not significant. Bold values identify the statistical significance in the comparison of two groups (p-value <0.05).
